# Combined Risk Assessment of Food-derived Coumarin with *in Silico* Approaches

**DOI:** 10.14252/foodsafetyfscj.D-21-00015

**Published:** 2022-09-23

**Authors:** Takashi Yamada, Naruo Katsutani, Taeko Maruyama, Tomoko Kawamura, Hiroshi Yamazaki, Norie Murayama, Weida Tong, Yasushi Yamazoe, Akihiko Hirose

**Affiliations:** 1 Division of Risk Assessment, Center for Biological Safety Research, National Institute of Health Sciences, 3-25-26 Tonomachi, Kawasaki-ku, Kawasaki 210-9501, Japan; 2 Showa Pharmaceutical University, Machida, Tokyo 194-8543, Japan; 3 National Center for Toxicological Research, Food and Drug Administration, 3900 NCTR Road, Jefferson, AR 72079, United States of America; 4 Division of Drug Metabolism and Molecular Toxicology, Graduate School of Pharmaceutical Sciences, Tohoku University, 6-3 Aramaki-Aoba, Aoba-ku, Sendai 980-8578, Japan

**Keywords:** coumarin, drug-induced liver injury score model, hepatotoxicity, physiologically based pharmacokinetics, individual susceptibilities

## Abstract

Hepatotoxicity associated with food-derived coumarin occurs occasionally in humans. We have, herein, assessed the data of existing clinical and nonclinical studies as well as those of *in silico* models for humans in order to shed more light on this association. The average intakes of food-derived coumarin are estimated to be 1−3 mg/day, while a ten-times higher level is expected in the worst-case scenarios. These levels are close to or above the tolerable daily intake suggested by a chronic study in dogs. The human internal exposure levels were estimated by a physiologically-based pharmacokinetic model with the use of virtual doses of coumarin in the amounts expected to derive from foods. Our results suggest that: (i) coumarin can be cleared rapidly *via* 7-hydroxylation in humans, and (ii) the plasma levels of coumarin and of its metabolite, *o*-hydroxyphenylacetic acid associated with hepatotoxicity, are considerably lower than those yielding hepatotoxicity in rats. Pharmacokinetic data suggest a low or negligible concern regarding a coumarin-induced hepatotoxicity in humans exposed to an average intake from foods. Detoxification of coumarin through the 7-hydroxylation, however, might vary among individuals due to genetic polymorphisms in CYP2A6 enzyme. In addition, the CYP1A2- and CYP2E1-mediated activation of coumarin can fluctuate as a result of induction caused by environmental factors. Furthermore, the daily consumption of food-contained coumarin was implicated in the potential risk of hepatotoxicity by the drug-induced liver injury score model developed by the US Food and Drug Administration. These results support the idea of the existence of human subpopulations that are highly sensitive to coumarin; therefore, a more precise risk assessment is needed. The present study also highlights the usefulness of *in silico* approaches of pharmacokinetics with the liver injury score model as battery components of a risk assessment.

## 1. Introduction

Coumarin is a naturally occurring organic chemical that is often ingested as part of cinnamon-containing foods. Although the intake of coumarin from foods is generally considered to be safe, coumarin-induced hepatotoxicity has been reported to occasionally occur in humans^1^^,^^2^^)^. Other than through the food intake, a clinical trial of coumarin has been performed on lymphedema patients, but the occurrence of hepatic disorders led to its withdrawal^3^^,^^4^^,^^5^^)^. The US Food and Drug Administration (FDA) has banned the use of coumarin as a food additive due to hepatotoxicity concerns.

The occurrence of hepatotoxicity has been observed in experimental animals such as dogs and rats after the administration of coumarin. The coumarin-induced hepatotoxicity is believed to be associated with the metabolism in the body. Coumarin is metabolized to *o*-hydroxyphenylacetic acid (*o*-HPA) through the reactive metabolite coumarin 3,4-epoxide^6^^,^^7^^)^, while the biological 7-hydroxylation is considered to be a process of detoxification. Species differences clearly exist between humans and rats in terms of the coumarin 7-hyroxylation. In fact, the detoxification rates are rapid in humans and slow in rats^8^^,^^9^^,^^10^^,^^11^^)^. Only CYP2A6 catalyzes coumarin 7-hydroxylation among the major cytochrome P450 enzymes in the human liver. This major detoxification pathway mediated by CYP2A6 is susceptible to genetic polymorphisms, but the influence of such genetic polymorphisms on the hepatotoxicity of coumarin still remains unclarified *in vivo* in humans^12^^)^. Moreover, multiple CYP enzymes participate in the 3,4-epoxidation, and the hepatic levels of these enzymes are known to vary among individuals. Therefore, an understanding of inter- and intra-individual differences in terms of their coumarin intake amounts and of their metabolic capacities is necessary in order to evaluate the risk of food-derived coumarin in humans.

In this study, human-relevant data on coumarin were collected, including data regarding its intakes from food, absorption, distribution, metabolism, and excretion (ADME), toxicity, and clinical information in an attempt to refine its risk assessment. Furthermore, two *in silico* models were introduced in order to provide additional human-relevant information: a physiologically-based pharmacokinetic (PBPK) model^11^^,^^13^^)^, and a drug-induced liver injury (DILI) severity-predicting model developed by the FDA (hereafter referred to as the FDA DILI score model)^14^^)^.

## 2. Materials and Methods

### 2.1 Intake, ADME and Toxicity Data Collection

Data on coumarin (including intake from food, experimental and clinical studies on the ADME and the *in vivo* and *in vitro* toxicity of coumarin) were obtained from the relevant regulatory assessment reports^1^^,^^15^^,^^16^^,^^17^^,^^18^^,^^19^^)^ and the literature.

### 2.2 PBPK Modeling

**Fig. 1.**
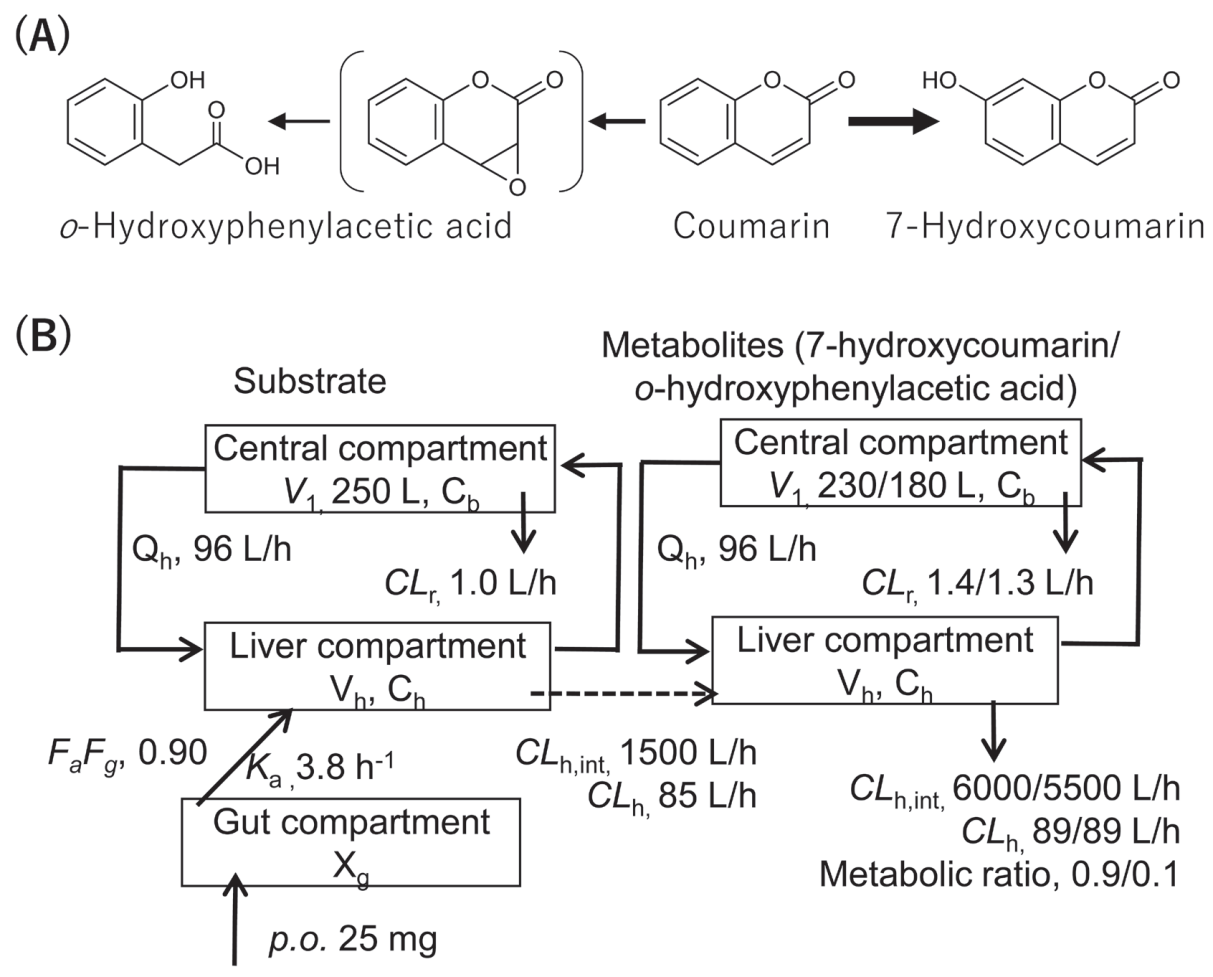
Major metabolic pathways of coumarin (A) and coumarin PBPK model (B). Input parameters for human coumarin PBPK model with two metabolites (7-hydroxycoumarin and *o*-HPA) were calculated previously^13^^)^. Values for fraction absorbed × intestinal availability (*F*_a_*F*_g_), absorption rate constant (*k*_a_), volume of the systemic circulation (*V*_1_), hepatic intrinsic clearance (*CL*_h,int_), hepatic clearance (*CL*_h_), and renal clearance (*CL*_r_) values for human PBPK models are shown. *X*_g_ represents the amount of compound in the gut compartment, *V*_h_ represents liver volume, *C*_h_ represents hepatic substrate concentration, and *C*_b_ represents blood substrate concentration.

The human PBPK models were constructed based on the rat PBPK model consisting of the gut, the liver, and the central compartments, as described previously^11^^)^. The blood concentration profiles in rats treated orally with 200 mg/kg of coumarin were reproduced^11^^)^. A scale-up strategy was applied by using the fixed values of the human body weight (70 kg) and the liver volume (1.5 L). The input parameters for the human PBPK model are presented in detail in Fig. 1. Systems of differential equations were used in order to obtain the concentrations of the substrate coumarin as well as those of the two metabolites (7-hydroxycoumarin and *o*-HPA) in the central compartments, as described previously^11^^)^.

### 2.3 Application of the FDA DILI Score Model for Coumarin and Related Drugs

Coumarin and related drugs sharing a substructure with coumarin (e.g., warfarin and methoxsalen) were applied to the FDA DILI score model^14^^)^. The DILI scores were calculated by using the following formula: DILI score = 0.608 × log_e_ (daily dose in mg) + 0.227 × logP + 2.833 (in the case of a present reactive metabolite formation). Prior to the application, the logP (the ratio of the concentration of the unionized compound at equilibrium between organic and aqueous phases) and the molecular weight (MW) were calculated by using the ADMET Predictor version 10.0 (SimulationsPlus, USA). Furthermore, the chemical classes of every substructure of coumarin were compared with those of the set molecules used for the construction of the model (a total of 354 human pharmaceuticals). The chemical classes of the substructures were searched in the public ChemoTyper application (Altamira LLC, USA and Molecular Networks GmbH, Germany). The chemical structures were profiled with the use of the OECD QSAR Toolbox^20^^)^ (ver. 4.4) for both coumarin and the set molecules.

## 3. Results

### 3.1 Collected data of Intake of Coumarin from Foods

In the European Food Safety Authority (EFSA) opinion of 2004, a theoretically-calculated maximum daily intake of coumarin was estimated to be 1.5 mg for an adult with a default body weight of 60 kg (0.025 mg/kg of body weight per day)^15^^)^. A mean coumarin intake from foods consumed within the Christmas season was estimated to be 5.0 mg per week, and the intakes of the heaviest consumers (six subjects) exceeded 35 mg/week^2)^. In Japan, a consumption of 2.45 g of cinnamon corresponding to the ingestion of 2.73 mg of coumarin from daily foods has been reported^21^^)^. The total amount of coumarin consumed can reach 5.18 mg/day if daily supplements that contain cinnamon are also consumed^21^^)^. In the worst-case scenarios, a food-mediated coumarin consumption of around 60 mg/day (based on a body weight of 50 kg) has been reported in Norway^18^^)^, and an additional exposure of 18 mg/day coumarin was calculated to occur from food supplements in Germany^16^^)^. These results suggest an average intake of approximately 1−3 mg of coumarin per day, as well as a level equal to ten-times higher the average intake in the worst-case scenario.

### 3.2 Collected data of Absorption, Distribution, Metabolism, and Excretion of Coumarin

In an *in vitro* intestinal epithelial cell monolayer system, a fraction-absorbed value of >0.9 was estimated for coumarin based on its apparent permeability. Coumarin is rapidly absorbed after an oral intake of 0.857 mg/kg, but its availability to the systemic circulation is reported to be less than 4%^13^^)^. The rest of the intake appeared in the form of 7-hydroxycoumarin and the glucuronide in the systemic-circulation, thereby suggesting an extensive first-pass effect. In a study employing an oral administration of 200 mg of coumarin in seven different subjects, 63.4% of the dose was recovered in the 24-h urine in the form of total 7-hydroxycoumarin^22^^)^. Coumarin can also be absorbed fairly efficiently after a dermal application, and the absorption rates ranged from 54.7% to 66.1% in humans^10^^)^.

The *in vivo* metabolic profiles of coumarin are similar among experimental animals and humans. Metabolites deriving from the oxidation of both the phenyl ring (7-hydroxylation) and the lactone ring (3,4-oxidation) can be detected mostly in the urine of mice, rats, dogs^10^^)^, and human volunteers^23^^)^; however, certain extents of a fecal excretion have also been observed in rats exposed to high doses^10^^)^.

7-Hydroxycoumarin and its glucuronic acid conjugate were the major metabolites detected in the urine of most individuals, while the lactone-ring opening metabolite, *o*-HPA, was slightly detected in urine; interestingly, the amount of *o*-HPA was more than that of 7-hydroxycoumarin in the 8-h urine of some individuals after a 2-mg coumarin intake^23^^)^. *o*-Hydroxyphenylacetaldehyde can be formed *in vitro* by microsomes from all four human liver samples as the major metabolite of coumarin at a coumarin concentration of 1 mM; however, 7-hydroxycoumarin was the major metabolite detected after an exposure to coumarin concentrations below 50 μM^24^^)^. These results suggest that both the coumarin concentration and the genetic background can affect coumarin metabolism in humans.

Studies of the toxicity mechanism of coumarin have consistently indicated a role for the reactive intermediate in coumarin-induced hepatotoxicity. The microsomal formation of metabolites bound covalently to hepatic proteins^8^^)^, the identification of *o*-hydroxyphenylacetaldehyde as a major metabolite of coumarin in the rat hepatic microsomes^8^^)^, the much lower toxicities of 3- or 4-methylcoumarin and of 3,4-dimethylcoumarin than coumarin^25^^)^, as well as the reactivity of *o*-hydroxyphenylacetaldehyde^26^^)^; all support the production of a reactive 3,4-oxide for the facilitation of the coumarin-mediated hepatotoxicity.

In human recombinant CYP systems, CYP1A1, CYP1A2 and CYP2E1 mediate the formation of *o*-hydroxyphenylacetaldehyde, and CYP3A4 may also support this reaction^27^^,^^28^^)^. The 7-hydroxycoumarin formation is supported only by CYP2A6, and no activities are detected with CYP1A1, CYP1A2, CYP2E1, and CYP3A4^28^^)^. CYP2A13 catalyzes both the 3,4-oxide formation and 7-hydroxylation of coumarin^29^^)^. The low levels of CYP2A13 are expressed selectively in extra-hepatic tissues, while the enzyme’s levels in the liver are negligible^30^^)^. Population studies suggest that 6% of the UK population is homozygous for the mutant CYP2A6 alleles, whereas the mutant CYP2A6 allele frequency may be as high as 48% in Japanese subjects^31^^)^.

The production of *o*-HPA was correlated with the CYP1A2 content of human hepatocytes^32^^)^. The production of *o*-HPA in the human liver microsomal system as well as *in vivo*, in the humanized-liver mice, was clearly inhibited in the presence of a selective inhibitor of CYP1A2, furafylline^33^^,^^34^^)^. These results suggest, at least partly, the involvement of CYP1A2 in the metabolic activation of coumarin in the human liver. CYP2E1 has also been expected to mediate the 3,4-epoxidation of coumarin in humans^27^^,^^35^^,^^36^^)^.

### 3.3 Collected data of Toxicity of Coumarin

Toxicity data of coumarin have been evaluated and published in the form of review articles^2^^,^^10^^,^^37^^,^^38^^)^ and risk assessment reports^1^^,^^15^^,^^16^^,^^17^^,^^18^^,^^19^^)^. These data consistently indicate the liver as the most sensitive target of coumarin in experimental animals. Hepatotoxicity in rats includes hepatic histopathological lesions along with increased liver enzymes at doses ≥50 mg/kg/day in a 2-year-long carcinogenicity study^19^^)^. Slight jaundice, marked histopathologic hepatic changes, and distended gall bladder were observed in a 1-year chronic study in dogs^39^^)^. In baboons, hepatic changes were limited to an increased liver weight and an hepatocyte endoplasmic reticulum hypertrophy observed at the highest coumarin dose (67.5 mg/kg/day) in a 2-year-long chronic toxicity study^40^^)^. The EFSA determined the tolerable daily intake (TDI) of coumarin to be 0.1 mg/kg/day, based on the no-observed-adverse-effect level (NOAEL) of 10 mg/kg/day found in a chronic toxicity study of coumarin in dogs, with an uncertainty factor of 100^15^^)^.

Hepatic disorder, characterized as an elevation of the liver enzyme levels, is the most common coumarin-associated adverse finding reported in humans^1^^,^^2^^)^. In one case, a 23-year-old woman was hospitalized with hepatitis after consuming 1–2 g of cinnamon (equivalent to 3.3–6.6 mg of coumarin) daily, for two months^2^^)^. According to the expert report on the assessment of coumarin in medicinal products^1^^,^^2^^)^, liver damage cannot be ruled out at a daily dose of 25 mg coumarin for a part of the population. In order to extrapolate from this effect level to a human NOAEL, a factor of 5 is considered as justified in the case of a severe effect at the lowest observed adverse effect level. Thus, an exposure to 5 mg of coumarin per day is expected to cause no adverse effects in sensitive subjects. Moreover, a TDI of 0.1 mg/kg body weight was derived^1^^,^^2^^,^^16^^)^. This value agreed well with the EFSA value based on animal data^15^^)^.

The incidence of coumarin-induced hepatotoxicity was estimated to be 0.37% by a clinical trial^41^^)^. In the National Institutes of Health LiverTox Database, the idiosyncratic, clinically apparent liver injury associated with coumarin was estimated to occur in 2 out of 1,000 patient-years of use^42^^)^.

### 3.4 Estimation of the Internal Exposure to Coumarin in Humans Using the PBPK Model

**Fig. 2.**
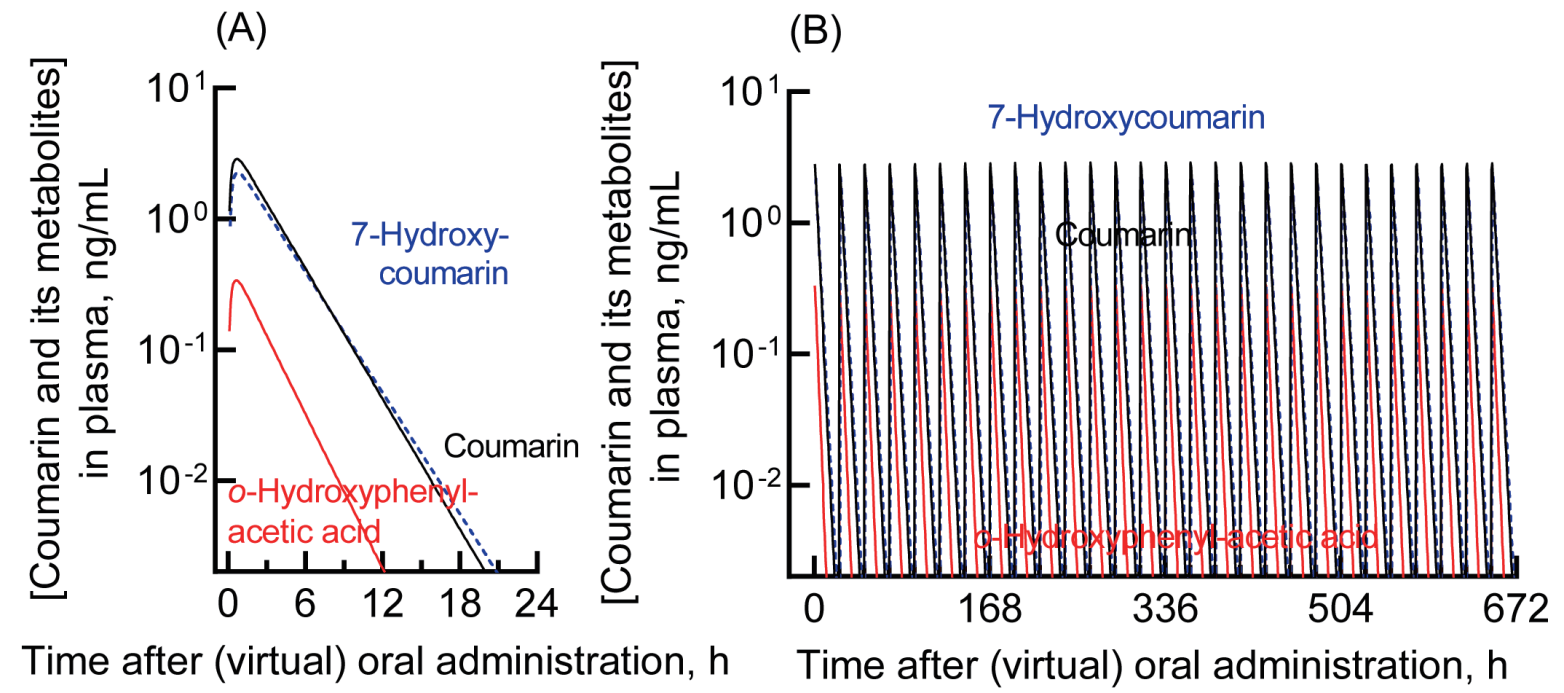
Plasma levels of coumarin and its metabolites in humans estimated using the established human PBPK model. The plasma concentrations of coumarin (black solid line), *o*-HPA (red solid line), and 7-hydroxycoumarin (blue dashed line) after virtual administration of 25 mg of coumarin via the oral route as a single dose (A) or daily doses for 28 days (B) are shown.

Pharmacokinetic data of coumarin in humans are necessary in order to assess the body exposure *in vivo* and the subsequent hepatotoxicity. However, the available data that are relevant to the assessment of possible toxic dose levels are limited^43^^)^. Therefore, the plasma concentrations of coumarin, 7-hydroxycoumarin, and *o*-HPA (generated *via* a coumarin 3,4-epoxidation) were estimated by using human PBPK models that have been previously developed and validated^11^^)^. Virtual oral doses of 2.5 mg and 25 mg were applied in the present study. The former dose corresponds approximately to the average intake of food-derived coumarin^21^^)^, and the latter dose corresponds to an amount close to the maximum estimate of coumarin intake from food or the amount of pharmaceutical administration that cannot exclude hepatotoxicity in a part of the human population^2^^)^. Plasma concentration curves after a single or a 28-day repeated virtual administration of coumarin in humans were generated by using a human PBPK model (Fig. 2). After a single dosing, the mean and the maximum plasma concentration after an intake of coumarin at a dose of 2.5 mg/day was estimated to be 0.04 and 0.29 ng/mL (0.27 and 2.0 nM), respectively. The results for the 25-mg oral dose are presented in Fig. 2A. The mean and the maximum plasma concentrations of coumarin after a single administration were 0.41 and 2.9 ng/mL (2.8 and 20 nM), respectively. The plasma concentration curves after the repeated virtual administration of coumarin in humans for 28 days were also generated by using the human PBPK model (Fig. 2B). The respective concentrations of coumarin, 7-hydroxycoumarin, and *o*-HPA after a repeated dosing were comparable to those after a single dosing, in consistent with the notion that coumarin is rapidly cleared and does not accumulate in the body.

### 3.5 Applicability to FDA DILI Score Model and DILI Scores

The possible hepatotoxicity of coumarin was evaluated by using a FDA DILI score model based on the daily dose of the substance, lipophilicity, and reactive metabolite formation^14^^)^.

**Fig. 3.**
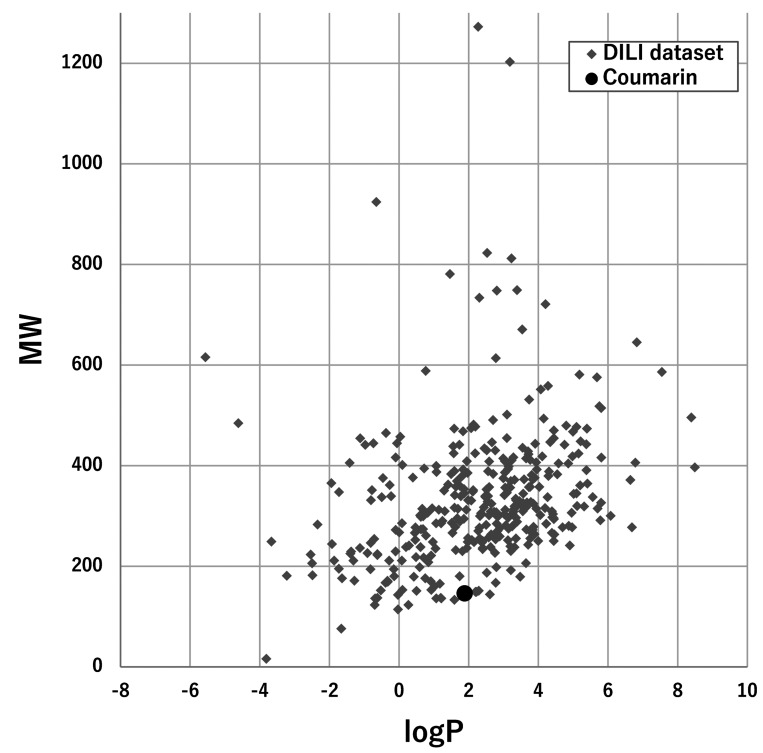
Application of coumarin to the FDA DILI score model. MW plotted against logP is presented for coumarin (black circle) and the set molecules for constructing the model, with a total of 354 substances (gray diamond).

The applicability of coumarin to the FDA DILI score model was assessed by plotting the logP and the MW of coumarin and the set molecules (Fig. 3). The estimated logP value of coumarin (1.39) was in the range of most of the set molecules (from −4 to 8), and the inclusion of the MW of coumarin (146.14) was also confirmed. Coumarin consisted of a total of 13 chemotypes (including benzopyrone), all of which were included in a set of chemotypes of the set molecules (Table 1 and Supplementary Data **Table S1**). The chemical structure profiling that was undertaken by using the OECD QSAR Toolbox yielded similar results (Supplementary Data **Table S2**).

**Table 1.** Chemotype of coumarin and duplication in the set of 354 molecules for constructing the FDA DILI score model

Chemotype contained in coumarin	No. of duplications in the set molecules
bond:C(=O)O_carboxylicEster_alkenyl	9
bond:C=O_carbonyl_generic	228
chain:alkeneCyclic_ethene_C_(connect_noZ)	37
chain:alkeneCyclic_ethene_generic	67
chain:aromaticAlkane_Ph-C1_cyclic	83
chain:aromaticAlkene_Ph-C2_cyclic	11
ring:aromatic_benzene	264
ring:hetero_[6]_O_pyran_generic	15
ring:hetero_[6]_Z_1-	100
ring:hetero_[6]_Z_generic	152
ring:hetero_[6_6]_O_benzopyran	3
ring:hetero_[6_6]_O_benzopyrone_(1_2-)	1
ring:hetero_[6_6]_Z_generic	52

Coumarin and the related drugs sharing a substructure with coumarin were applied to the FDA DILI score model (Table 2). The epoxidation of coumarin and of methoxsalen were assumed to lead to the formation of reactive intermediates^15^^,^^16^^)^. The DILI scores of coumarin were calculated to be 3.71 and 5.11 for the daily consumption levels of 2.5 and 25 mg/day, respectively, and the risk was judged to be moderate (score of 3–6). Warfarin (10 mg/day) was judged to be of low risk based on a DILI score of 1.95, whereas methoxsalen (3 mg/day) was considered to be of medium risk based on a DILI score of 3.92.

**Table 2.** Application of coumarin and related drugs to the FDA DILI score model

Chemicals	Daily Dose (mg/day)	logP	RM formation	DILI score	DILI risk	Remarks
Coumarin 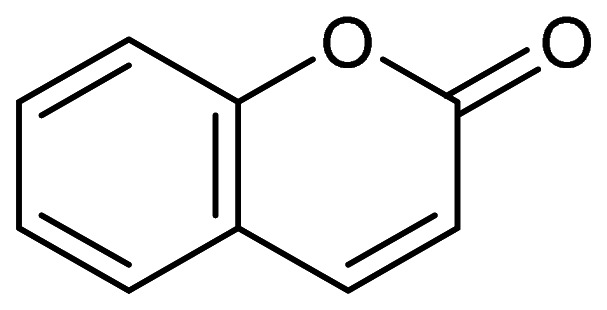	2.5	1.39	Yes (1)	3.71	M	Daily dose as food with very occasional elevated liver enzymes
	25			5.11	M	Daily dose as medicine for lymphedema and occasionally taken as food with occasional elevated liver enzymes
Warfarin 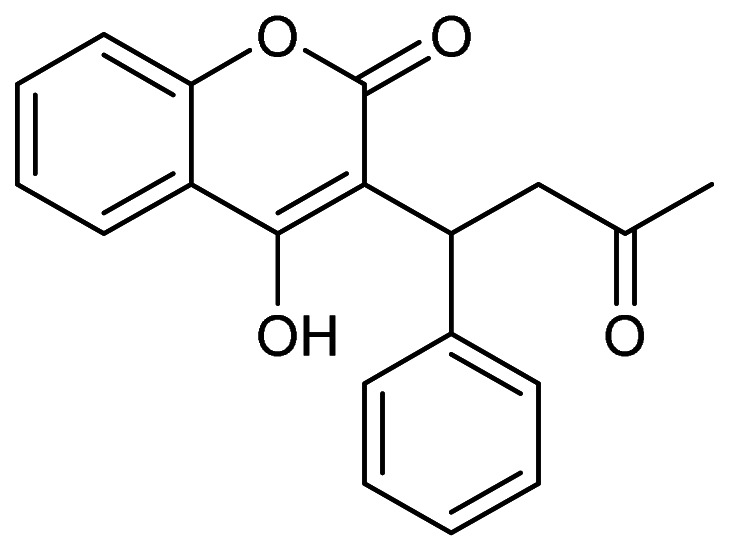	2	2.44	No (0)	0.98	L	Daily dose as medicine (anticoagulant) with rare hepatotoxicity cases
	10			1.95	L	
Methoxsalen 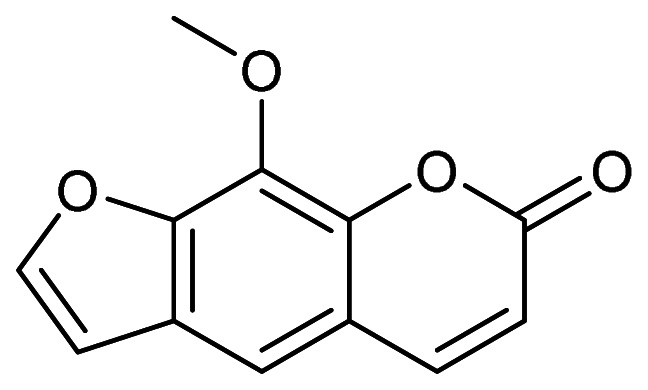	3	1.93	Yes (1)	3.92	M	Daily dose as medicine for psoriasis with occasional elevated liver enzymes (2%–12% of patients)
	40			5.51	M	

DILI score based on daily dose, lipophilicity (logP), and presence (1) or absence (0) of reactive metabolite (RM) formation was calculated following the formula: DILI score = 0.608 × log_e_(daily dose by mg) + 0.227 × logP + 2.833 × 1/0. DILI risk was classified according to DILI score as low (L, <3), moderate (M, 3–6), and high (H, >6)

## 4. Discussion

In this study, the hepatotoxicity of food-derived coumarin in humans was comprehensively reevaluated by the use of the data of existing clinical and animal studies as well as those of generated by us through *in silico* approaches for humans.

According to several studies, the average intake of food-derived coumarin is about 1−3 mg/day, and in the worst-case scenarios, the intake is expected to be about ten-times higher than that of the average amount. The levels were found to be almost equal to or above the TDI (0.1 mg/kg/day) that derived from animal studies^15^^)^.

It has been shown that the majority of coumarin is rapidly converted to 7-hydroxycoumarin, along with low levels of *o*-HPA which is associated with the metabolic activation (Fig. 2A). At a virtual intake of 25 mg of coumarin (that is approximately ten-times more than the average intake), the maximum plasma concentrations of coumarin and *o*-HPA were expected to be 20 nM and 2 nM, respectively, at 0.7 h (Fig. 2A). Based on the plasma-to-liver distribution ratios of coumarin and *o*-HPA of 0.875 and 0.504^11^^)^, the coumarin and *o*-HPA concentrations in the liver at the same timepoint were estimated to be 17 nM and 1.1 nM, respectively.

The maximum blood concentrations of coumarin and *o*-HPA were estimated to be approximately 200 μM and 80 μM, respectively, at 0.5 h after the administration of a toxic dose of 200 mg/kg^44^^)^. Therefore, clear differences were observed with regard to both the coumarin and the *o*-HPA levels between the simulated data (at 25 mg/kg) in humans and measured data (at 200 mg/kg) in rats. Based on the pharmacokinetics data, an intake of 25 mg/kg of coumarin in humans may be considered to be of low or subtle concern for the development of hepatotoxicity, as far as the human population maintains average levels of capacity for coumarin metabolism. It should be noted, however, that this PBPK model is based on data deriving from a small number of healthy individuals. The report by Abraham et al. in 2010^2^^)^ claims that hepatotoxicity concerns cannot be ruled out in humans after the ingestion of 25 mg or more of coumarin. These results suggest the existence of a subpopulation that is highly susceptible to coumarin-induced hepatotoxicity. Further investigation of such individual differences in terms of the susceptibility to coumarin is warranted.

The detoxification of coumarin in humans is mainly mediated by CYP2A6^32^^,^^35^^,^^45^^)^, which may imply variations in the susceptibility to coumarin toxicity as a result of genetic polymorphisms in the metabolizing enzyme^46^^,^^47^^,^^48^^)^. The polymorphism of CYP2A6 is rather prevalent in the Japanese population, and the non-wild (poor metabolizer) types were reportedly present in nearly half of the population. In addition, both CYP1A2 and CYP2E1 mediate the production of *o*-hydroxyphenylacetaldehyde, which is probably associated with coumarin toxicity. The hepatic levels of CYP1A2 and CYP2E1 vary under the influence of various environmental factors. Cigarette smoking and alcohol consumption are known to alter the hepatic levels of CYP1A1/2 and CYP2E1 through induction phenomena, respectively. Other dietary components are also known to modulate the activation and the detoxification of coumarin through the processes of inhibition and transport. Therefore, further studies on the impact of individual differences are required in order to refine the evaluation of the coumarin-induced hepatotoxicity in humans.

The FDA continues to compile human hepatotoxicity data of approved or withdrawn drugs, and these data include their daily dose, their lipophilicity, and their reactive metabolite formation. These data are also used for the construction of the QSAR model aiming to predict the severity of clinical liver injury^14^^)^. Available toxicity data of foods and food ingredients are often not sufficient for the rigid evaluation of their toxicities in humans and, thus, the use of this model is expected to be beneficial. The applicability was at first checked by the comparison of the logP, the MW, and the chemotypes of coumarin with those of a total of 354 molecules that were used in order to construct the model. Coumarin was judged to be applicable, and the daily intakes of coumarin at 2.5 and 25 mg/kg were predicted to be of moderate risk. The structurally related drug, warfarin, was predicted to be of low risk, whereas methoxsalen was predicted to be of moderate risk. The estimated results of the coumarin-related drugs are consistent with hepatotoxicity in humans, thereby offering information on a relative probability for the development of coumarin-induced hepatotoxicity. The DILI score model may also be applicable to food ingredients other than coumarin for the preliminary discrimination or evaluation of potential hepatotoxicity in humans.

In summary, the possibility of developing coumarin-induced hepatotoxicity in humans was, herein, reevaluated through a combined approach that integrated the existing data of clinical and animal studies with data deriving from *in silico* models. At the current average coumarin intake, the humans can be considered to be safe, at least as far as the coumarin-induced hepatotoxicity is concerned. On the other hand, the existence of a human subpopulation that is highly susceptible to the hepatotoxicity of coumarin is suggested. Further studies are required in order to achieve a more precise risk assessment that would take into account the individual differences in coumarin metabolism as defined by genetic and environmental factors. Moreover, the present study highlights the usefulness of *in silico* approaches of pharmacokinetics and the liver injury score model as battery components of a risk assessment.

Disclaimer

This manuscript reflects the views of the authors and does not necessarily reflect those of the National Institute of Health Sciences and the US Food and Drug Administration. Any mention of commercial products is for clarification only and is not intended as approval, endorsement, or recommendation.
